# Urine retention as presenting manifestation of tuberculous meningitis complicated by lacunar infarction and transverse myelitis: Case report and literature review

**DOI:** 10.1002/ccr3.4489

**Published:** 2021-07-23

**Authors:** Ahmed Salih Abdulhadi, Elabbass A. Abdelmahmuod, Jawaher Al‐Rubaiei, Elrazi Ali, Ahmed Emad Mahfouz, Amer Farooqi

**Affiliations:** ^1^ Department of Internal Medicine Hamad Medical Corporation Doha Qatar; ^2^ Qatar University Doha Qatar; ^3^ Clinical Imaging Department Hamad Medical Corporation and Weill Cornell Medical School in Qatar Doha Qatar

**Keywords:** cerebral infarction, tuberculous meningitis, lacunar infarct, corticosteroid

## Abstract

Early diagnosis and management of tuberculous meningitis will prevent lethal and fatal neurological complications such as acute infarction and permanent disability.

## Graphical abstract

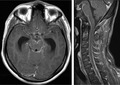

## 1 INTRODUCTION

Complications of tuberculous meningitis in the cerebrovascular system are widespread and represent its most serious legacy. In tuberculous meningitis (TBM), cerebral vessel damage and brain infarctions have long been documented. They have been under extensive pathological study since the late nineteenth and early twentieth centuries. They are the primary cause of permanent brain damage in TBM, and their most significant effect is widely regarded. In clinically diverse ways, they are present and continue to develop throughout the initial stages of treatment. Magnetic resonance imaging is an imaging technique of choice for identifying brain infarctions, usually displaying numerous or bilateral lesions in the areas of the perforating vessels of the middle cerebral artery. We reported a 29‐year‐old man who presented with urine retention and confusion diagnosed with tuberculous meningitis, which was later complicated with asymptomatic acute lacunar infarction and transverse myelitis, and he was started on antituberculous medications. He improved and was discharged on anti‐TB medications. The widespread understanding of the significance of early diagnosis and timely care of TBM illustrates the need to avoid cerebrovascular complications, at least in part.

Tuberculous meningitis (TBM) stroke occurs in 15%–57% of patients, especially in the disease's advanced and extreme stages. Because of being in a quiet area or in a deep coma or related pathology such as spinal arachnoiditis or tuberculoma, most strokes can be asymptomatic.^1^

Despite the availability of successful antituberculosis (anti‐TB) medications, tuberculous meningitis (TBM) is potentially lethal with risks of serious neurological sequelae.^2^ Elevated intracranial pressure, cranial neuropathies, optic neuropathy, hydrocephalus, epilepsy, hypopituitarism, spinal arachnoiditis, myelopathy, and radiculopathy are complex neurological complications of TBM.^3^

The inflammatory exudate in TBM is formed within the subarachnoid cisterns. Exudative basal meningitis strengthens strangulation, spasm, constriction, periarteritis, and even necrotizing panarteritis of vessels of Willis' circle develop, with or without secondary thrombosis. These modifications decrease the flow of arterial blood, causing ischemia and cerebral infarction.^4^

The pathology of the vessel tends to derive from its immersion in the local inflammatory exudate. Pathologies of infiltrative, proliferative, and necrotizing vessels have been identified, but the relative contributions to brain damage of each luminal thrombosis remain uncertain.^5^

There is some evidence that strokes can be mediated by vasospasm early in the disease and subsequent strokes by proliferative intimal disease. In avoiding vascular complications, antituberculous medications tend to be relatively unsuccessful, possibly indicating an immune mechanism. A preventive function for corticosteroids, however, is still to be proven.^6^

## 2 CASE PRESENTATION

A 29‐year‐old man with no past medical history presented to the emergency department with lower abdominal pain and urine retention, and abnormal behaviors lasting 2 days. He was admitted to the hospital, and a Foley catheter was inserted and 500 ml was drained. Bedside ultrasound showed mild bilateral hydronephrosis, and pelvic ultrasound was normal. He did not improve; later, he developed high‐grade fever, confusion, photophobia, and inability to walk. On examination, there was a high‐grade fever of 39.5, neck stiffness, and positive Kernig's and Brudziński's signs.

There was no back tenderness or deformity. However, X‐ray and MRI of the back were normal.

Complete blood count (CBC) was sent and showed (see Table 1) high white blood cell (12,000) and anemia (Hb 10.9).

**TABLE 1:** Complete blood count

Detail	Value w/units	Normal range	Flags
WBCs	12.7 × 10^3^/μl	4.0–10.0	High
Absolute neutrophil count (ANC)	9.6 × 10^3^/μl	2.0–7.0	High
Hgb	10.9 g/dl	13.0–17.0	Low

CT head showed mild dilatation of the supratentorial ventricular system with no evidence of obstructive lesion.

Lumbar puncture was done to rule out bacterial meningitis, and the result was consistent with TB meningitis (see Table 2), despite TB AFB, PCR, and culture being negative.

**TABLE 2:** Result of LP which is consistent with TB meningitis

Detail	Value w/units	Normal range	Flags
Color CSF	Xanthochromic		
Appearance CSF	Turbid		
WBC CSF	333/μl	0–5	High
RBC CSF	26/μl	0–2	High
Neutrophils CSF	13%	0–6	High
Lymphocyte CSF	81%	40–80	High
Monocyte CSF	6%	15–45	Low
CSF glucose	2.87 mmol/L	2.22–3.89	
CSF protein	>6.00 g/L	0.15–0.45	Very high

**FIGURE 1:**
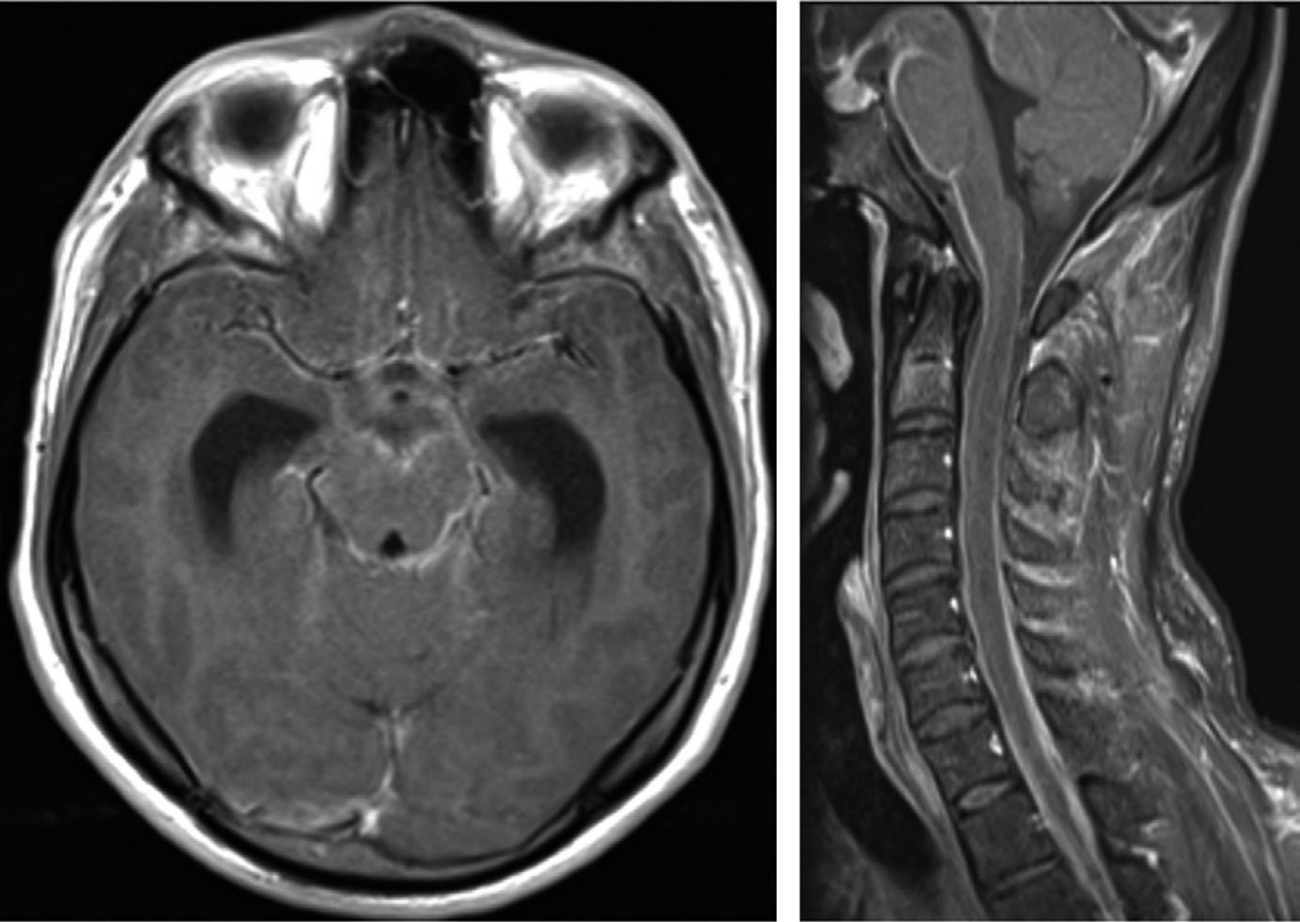
Dilatation of the cerebral ventricles and abnormal enhancement of the meninges at the posterior fossa and the spinal canal consistent with meningitis

**FIGURE 2:**
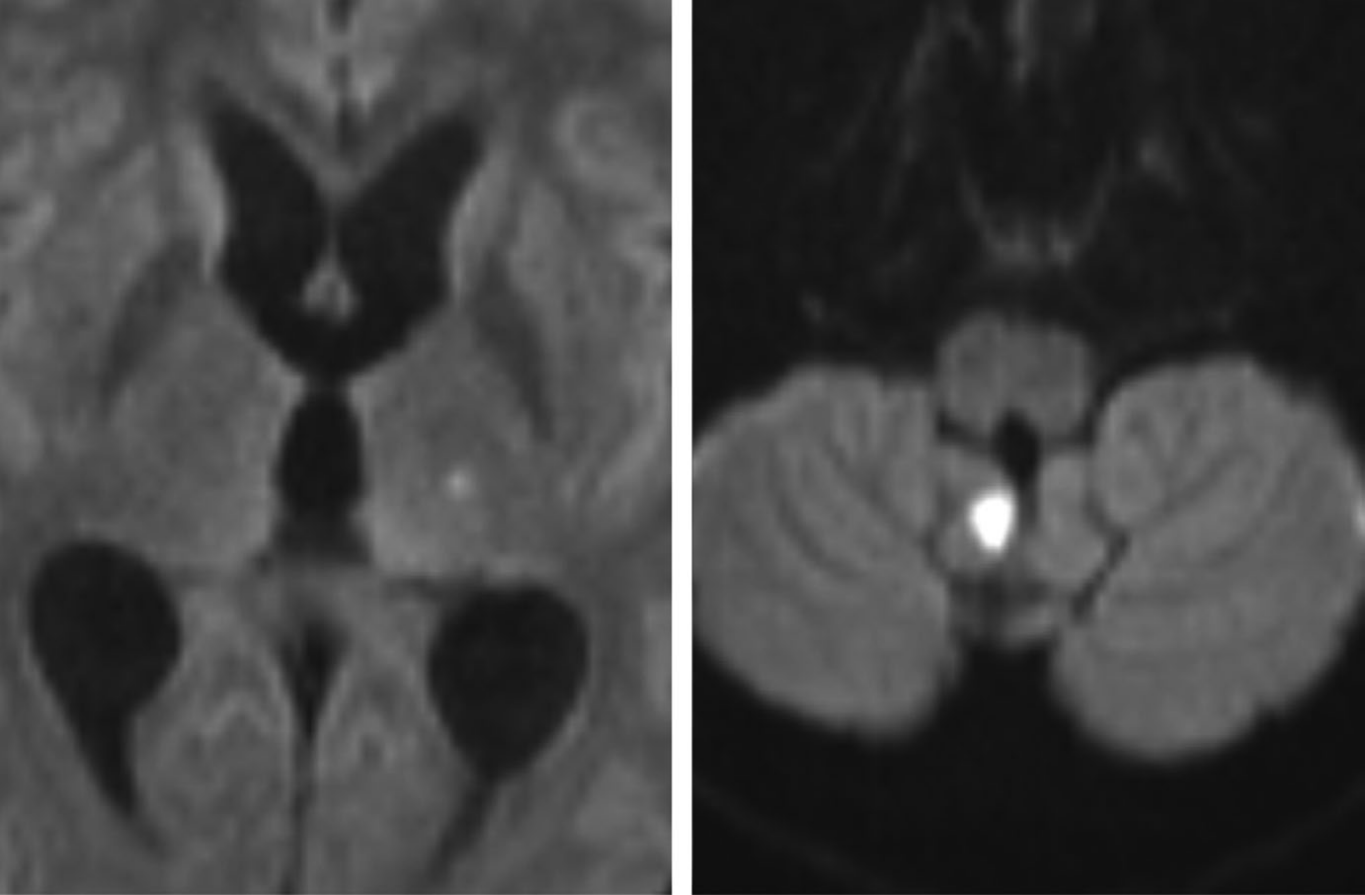
Gadolinium‐enhanced MR images of the head and cervical spine demonstrate dilatation of the cerebral ventricles

**FIGURE 3:**
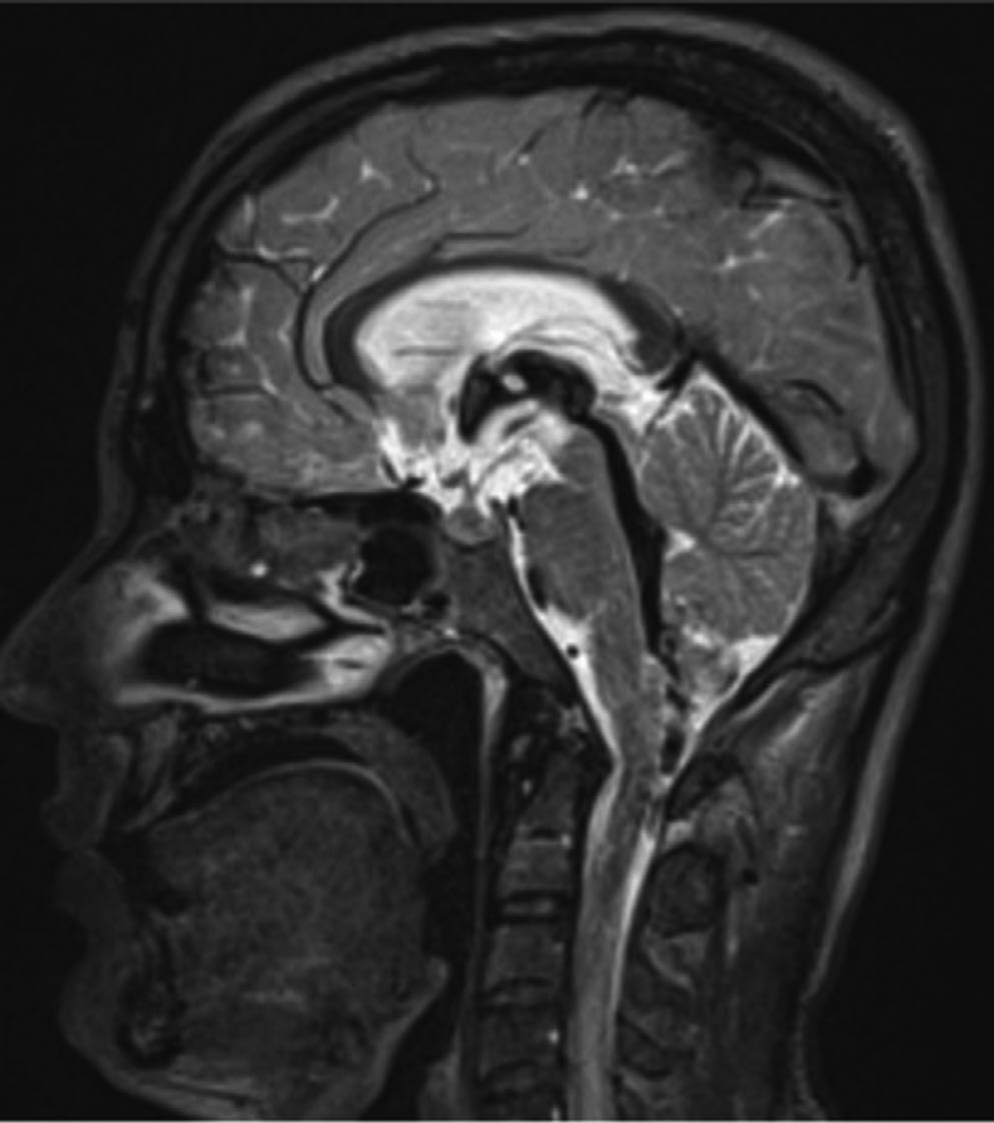
Sagittal T2‐weighted MR image demonstrates altered signal intensity of the spinal cord, dilatation of the cerebral ventricles

He was started empirically on ceftriaxone (2‐g BID for 14 days), dexamethasone (8 mg q6 h), and anti‐TB medications (isoniazid, rifampicin, ethambutol, and pyrazinamide). During the hospital stay, an MRI head was taken (see Figures 1, 2, 3) which showed lacunar infarction and transverse myelitis.

He was continued on anti‐TB medications, and the patient showed significant improvement. He was able to walk, regained his consciousness, and was able to pass urine. The patient was discharged with prednisolone tapering and anti‐TB medications with follow‐ups in the TB clinic to complete anti‐TB for 12 months.

## 3 DISCUSSION

With a huge and rising worldwide burden, tuberculosis is a major health concern. The worldwide tuberculosis estimates for 2007 were 9.27 million new cases and 1.78 million deaths. A correlation between tuberculosis, atherosclerosis, and stroke that sometimes corresponds to tuberculosis of the central nervous system (CNS) has been suggested.^7^

Intense basal exudative meningitis can lead to vasculitis around Willis's circle, leading to cerebral infarction (CI) which may be asymptomatic or symptomatic as ischemic stroke. Cerebral infarction is the greatest permanent disability risk factor.^8^

Hypersensitive response is thought to be one of the causes of arteritis in tuberculous meningitis. There is no awareness of preventive measures and the best therapies for cerebral infarction complicating tuberculous meningitis. We looked up for those patients' clinical and radiological data to gain insights into possible preventive measures for this extreme neurological complication of tuberculous.^9^

It was thought that corticosteroids with antituberculosis therapy decreased mortality and morbidity, but their function in reducing strokes has not been demonstrated. Aspirin also decreases mortality, and further studies are needed to prove the efficacy in reducing stroke in TBM.^10^

Tuberculous meningitis vascular complications are widespread and are related to adverse outcomes. Mortality in TBM patients with infarction is three times higher than in that in patients without infarction. The level of vascular involvement and cerebral infarction is the key determinants of prognosis among survivors. Kids with big, multiple, and bilateral infarctions have the worse motor and neurodevelopmental outcomes.^11^

There is no definite role of vessel thrombosis in causing cerebral infarction in TBM. Some authors have either struggled to find arterial thromboses or discovered them to be rare when explicitly sought in autopsy TBM brains.^12^

In comparison, some have indicated that thrombosis is significant in causing venous thrombosis or that spinal artery thrombosis has been emphasized, while others argue that thrombosis is typically relatively normal in TBM in connection with vasculitis.^13^

There were few cases reported and meta‐analyses that link the association between tuberculous meningitis and showed that stroke presence has been considered a bad prognosis of TB meningitis.^14^

In a survey of 25 children with infarction‐related TBM, no one has completely recovered.^15^

In another study of 40 patients with TBM, despite the addition of dexamethasone therapy, two thirds of patients with cerebral infarction complications had a poor outcome at 3 months of treatment.^16^

Urinary retention is common, and urologists treat the majority of patients who have it. Patients are given the necessary tests to rule out common causes of urinary retention, such as urethral stricture or prostatic enlargement. There could be a physiological cause if there is no systemic urological lesion.^17^

The presence of neurological deficits in the lower limbs may indicate a spinal or nerve root lesion. When the neurological examination is normal, the diagnosis is dependent on further testing.^18^

In our patient, the most common causes were excluded, like urethral stricture and prostatic enlargement, and after initiation of anti‐TB medications and steroids, his symptoms subsided.

## 4 CONCLUSION

The widespread understanding of the significance of early diagnosis and timely care of TBM illustrates the need to avoid cerebrovascular complications, at least in part. We have concentrated especially on pathological and etiopathogenic aspects in this case report and literature review of tuberculous cerebrovascular disease, in the hope that this may encourage more research into fair and targeted intervention.

## CONFLICT OF INTEREST

None declared.

## AUTHOR CONTRIBUTIONS

ASA: Writing, editing, and final approval. EAA: Literature review, writing, editing, and final approval. JA‐R: Writing. EA: Literature review and writing. AEM: Imaging review. AF: Literature review and writing.

## ETHICAL APPROVAL

This case was approved by the Hamad Medical Corporation's Medical Research Center, and written informed consent was obtained from the patient for publication of this case report and any accompanying images.

## Data Availability

The data that support the findings of this study are available from the corresponding author upon reasonable request.
